# Motive-Oriented, Personalized, Internet-Based Interventions for Depression: Nonclinical Experimental Study

**DOI:** 10.2196/37287

**Published:** 2022-09-13

**Authors:** Lara Bücker, Thomas Berger, Alina Bruhns, Stefan Westermann

**Affiliations:** 1 Department of Psychiatry and Psychotherapy University Medical Center Hamburg-Eppendorf Hamburg Germany; 2 Department of Clinical Psychology and Psychotherapy University of Bern Bern Switzerland; 3 Medical School Hamburg University of Applied Sciences and Medical University Hamburg Germany; 4 Department of Psychology University of California Berkeley, CA United States

**Keywords:** internet-based interventions, depression, adherence, motive orientation, personalization

## Abstract

**Background:**

The low level of adherence in internet-based self-help interventions for depression suggests that in many existing programs, the motivational fit between the program and the user is unsatisfactory (eg, the user seeks autonomy, but the program provides directive guidance). Personalized, motive-oriented, self-help interventions could enable participants who interact with a program and its contents to have more engaging and less aversive experiences and thus increase adherence.

**Objective:**

In an experimental study with a nonclinical analogue sample, we aimed to test the hypotheses that a better motivational person-program fit is linked with higher anticipated adherence, working alliance, and satisfaction with the program.

**Methods:**

Motivational person-program fit was examined with respect to the 2 contrasting motives *being autonomous* and *being supported*. The hypotheses were tested by specifically varying the motivational person-program fit in a nonclinical sample (N=55), where participants were asked to work on, and subsequently evaluate, a limited set of individual pages of a self-help program with guidance (in the form of text messages) for depression. The sections of the self-help program were redesigned to either particularly address the autonomy motive or the support motive. For the quasi-experimental variation of the motivational person-program characteristics, we divided the 55 participants into 2 groups (autonomy group: n=27, 49%; support group: n=28, 51%) by screening method (using the Inventory of Approach and Avoidance Motivation), corresponding to the 2 motives. Both groups evaluated (in randomized order) 2 excerpts of the program—one that matched their motive (fit) and one that was contrary to it (no fit). Immediately after the evaluation of each excerpt, anticipated adherence, working alliance, and treatment satisfaction were assessed.

**Results:**

Regarding *being supported*, the satisfaction with or violation of this motive had an impact on (optimal) anticipated adherence as well as working alliance and satisfaction with the intervention; a congruent person-program fit resulted in significantly higher anticipated adherence (*t*_27_=3.00; *P*=.006), working alliance (*t*_27_=3.20; *P*=.003), and satisfaction (*t*_27_=2.86; *P*=.008) than a noncongruent fit. However, a similar impact could not be found for the motive *being autonomous*. Several correlations were found that supported our hypotheses (eg, for the congruent person-program fit autonomy motive and autonomy group, support satisfaction negatively correlated with optimal anticipated adherence).

**Conclusions:**

This first experimental study gives reason to assume that motive orientation may have a positive influence on adherence, working alliance, and satisfaction in internet-based self-help interventions for depression and other mental disorders. Future studies should conduct randomized controlled trials with clinical samples and assess clinical outcomes.

## Introduction

### Background

To address the gap in the treatment of mental disorders [1], in the last decade, numerous internet-based interventions have been developed and investigated with regard to feasibility, acceptance, effectiveness, and side effects [2-4]. Internet-based interventions have some well-known advantages (eg, higher flexibility, resource saving, applicability, lower costs, and high levels of anonymity) over conventional treatment options such as psychotherapy and are therefore assumed to be able to address specific treatment barriers and narrow treatment gaps [5-7]. Internet-based interventions can be categorized as either self-guided (no additional individual therapeutic support), guided (with individual therapeutic support in the form of messages or contact through telephone), or blended (combination of internet-based programs and conventional psychotherapy) [8-10]. Most of these programs have been developed for anxiety disorders and depression [11], although programs for other mental disorders such as schizophrenia or pathological gambling are also addressed [12,13]. For programs with therapeutic guidance, meta-analyses have found effect sizes comparable to those for conventional face-to-face psychotherapy [2,7]. For self-guided programs, the effects reported are smaller; however, there are several advantages that speak for their use (eg, they provide increased access to treatment for those who do not meet the full criteria of a disorder, are cost-effective and resource saving, and there are no waiting times [14,15]).

### Adherence in Internet-Based Interventions

Although internet-based interventions have proven effective in reducing psychological symptoms, treatment adherence in such interventions is usually low, and treatment dropout rates are high [15,16]. Rates of dropout from internet-based interventions for depression can be as high as 75%, with a mean of 32% (SD 17%) [17]. Several predictors of treatment adherence in self-guided internet-based interventions for depression have been identified: male gender, low educational background, and comorbid anxiety disorders are associated with low adherence [18]. A more recent study found that a higher educational level, extraversion (personality trait), and participants’ use of cognitive behavioral therapy skills predicted lower dropout risk, whereas technical difficulties and openness to experience predicted higher dropout risk [17]. Studies consistently found that only a minority of participants use self-guided interventions on a regular basis [13,16,19-21]. Findings further indicate that use is associated with effectiveness: those who regularly (ie, several times a week) work with the interventions do benefit best in terms of symptom reduction [22,23]. It is therefore crucial to investigate ways on how to enhance users’ motivation to use such interventions frequently. Although Titov et al [23] found that sending reminders through automated emails facilitated treatment outcomes, a previous study on a smartphone app self-help intervention for depression aimed to increase use by sending daily smartphone reminders through push notifications but still found frequent use only for a few participants [22]. Consequently, it is necessary to look for new approaches that can sustainably increase adherence, possibly by personalizing interventions. Attempts to date to increase adherence are invariably based on nontheoretical trials, and there is a lack of studies that test theoretically based approaches embedded in basic research.

### Personalization to Increase Adherence

There is widespread agreement that all forms of psychological interventions should be tailored to individual patient characteristics and needs [24]. We hypothesize that by adapting the content of internet-based interventions to specific patient characteristics as well as user needs and motives, adherence to these interventions can be increased. According to a recent meta-analysis, adapting psychotherapy to specific patient preferences is associated with fewer treatment dropouts and more positive treatment outcomes than providing a nonpreferred type or mode of treatment [25]. Another meta-analysis investigated the extent to which treatment outcomes are improved when therapists offer less directive treatments to patients with high reactance [26,27]. The results showed a large effect size (Cohen *d*=0.79) and confirmed that individuals with high reactance had better outcomes when therapists adopted a reflective and nondirective attitude rather than a directive and authoritarian one. To a slightly lesser extent, a directive and authoritarian attitude for individuals with low reactance did yield better outcomes too.

When personalizing internet-based interventions, it is crucial to look beyond symptom severity [28]. Furthermore, personalization should not only mean that users receive personalized feedback on their exercises, but rather that the contents and elements of self-help programs are adapted, possibly based on a preliminary assessment. Berger et al [29] evaluated an internet-based, tailored, guided self-help treatment for social anxiety disorder, panic disorder with or without agoraphobia, and generalized anxiety disorder by comparing the tailored treatment with both a standardized disorder-specific internet-based treatment and a wait-list control group. The study found large effect sizes for both active groups as well as the control group (Cohen *d*=0.80 and Cohen *d*=0.82, respectively), but no difference was found in effectiveness in all outcomes between the 2 active groups. Furthermore, a Swedish research group developed internet-based interventions for anxiety disorders and depression that tailor the content to the individual symptoms of the user and evaluated the approach in several trials [30-32]. The group found that the tailored approach was superior to the approaches used for the active control groups (web-based discussion group [30] and standardized, nontailored internet-based treatment [32]). A more recent study compared a tailored internet-based intervention for depression and anxiety in older adults with weekly general support and found a higher reduction of anxiety (Cohen *d*=0.50) in the tailored approach [33]. Moritz et al [34] evaluated a web-based self-help program for obsessive-compulsive disorder and examined whether a version tailored to individual problems would produce greater effects than the full program, but they did not find better outcomes for the tailored approach. In most of these programs, tailoring consisted of selecting and sequencing existing modules specifically for the user based on information obtained from a web-based assessment. Another option could be to adapt internet-based interventions to individual motives of users [35].

### Motive-Oriented Internet-Based Interventions

According to the *consistency theory*, human functioning can be understood as need driven, and approach and avoidance motives are assumed to promote satisfaction of the basic needs for control and orientation, pleasure, attachment, and self-enhancement, as well as to prevent frustration or violation of these needs [36-38]. In this context, *incongruence* can be understood as insufficient motive satisfaction in interaction with the environment [39]. In psychotherapy, therapists derive the most important motives of their patients from their behavior (*bottom-up*) with the help of plan analysis and proactively take them into account when building and maintaining the therapy relationship [40].

From a motivational perspective, internet-based interventions can be understood as environments that individuals access to satisfy their individual needs or to prevent the needs from being violated [35,36]. The low level of adherence in self-guided internet-based interventions for depression suggests that in many existing programs the motivational fit between the program and the user is unsatisfactory (eg, the user seeks autonomy, although the program provides much support and guidance in a directive style). Personalized, motive-oriented, self-guided interventions could enable individuals who interact with the program and its contents to have more engaging and less aversive experiences and thus increase treatment adherence [41,42].

### Objectives of This Study

Within a nonclinical experimental study, excerpts of a web-based intervention for depression were adapted to the following 2 potentially opposing motives: *being autonomous* and *being supported*. Using these 2 motives, we tested the hypotheses that a better motivational person-program fit is associated with (1) higher anticipated adherence, (2) working alliance, and (3) satisfaction with the program.

## Methods

### Study Design

This study was based on an experimental design. The independent variables were (1) group (groups screened with the Inventory of Approach and Avoidance Motivation based on terciles: autonomy group [AutGrp]=autonomy high, >2/3, and support low, <1/3, vs support group [SuppGrp]=support high, >2/3, and autonomy low, <1/3) and (2) motive orientation through 2 versions of excerpts of a self-help internet-based intervention (autonomy condition [AutCond] and support condition [SuppCond]) tailored to the 2 groups. A motivational fit existed in the conditions AutCond-AutGrp and SuppCond-SuppGrp, whereas a discrepancy existed in the conditions SuppCond-AutGrp and AutCond-SuppGrp. The dependent variables were motivational incongruence during program use, expected adherence, working alliance, and treatment satisfaction.

### Procedures

**Figure 1:**
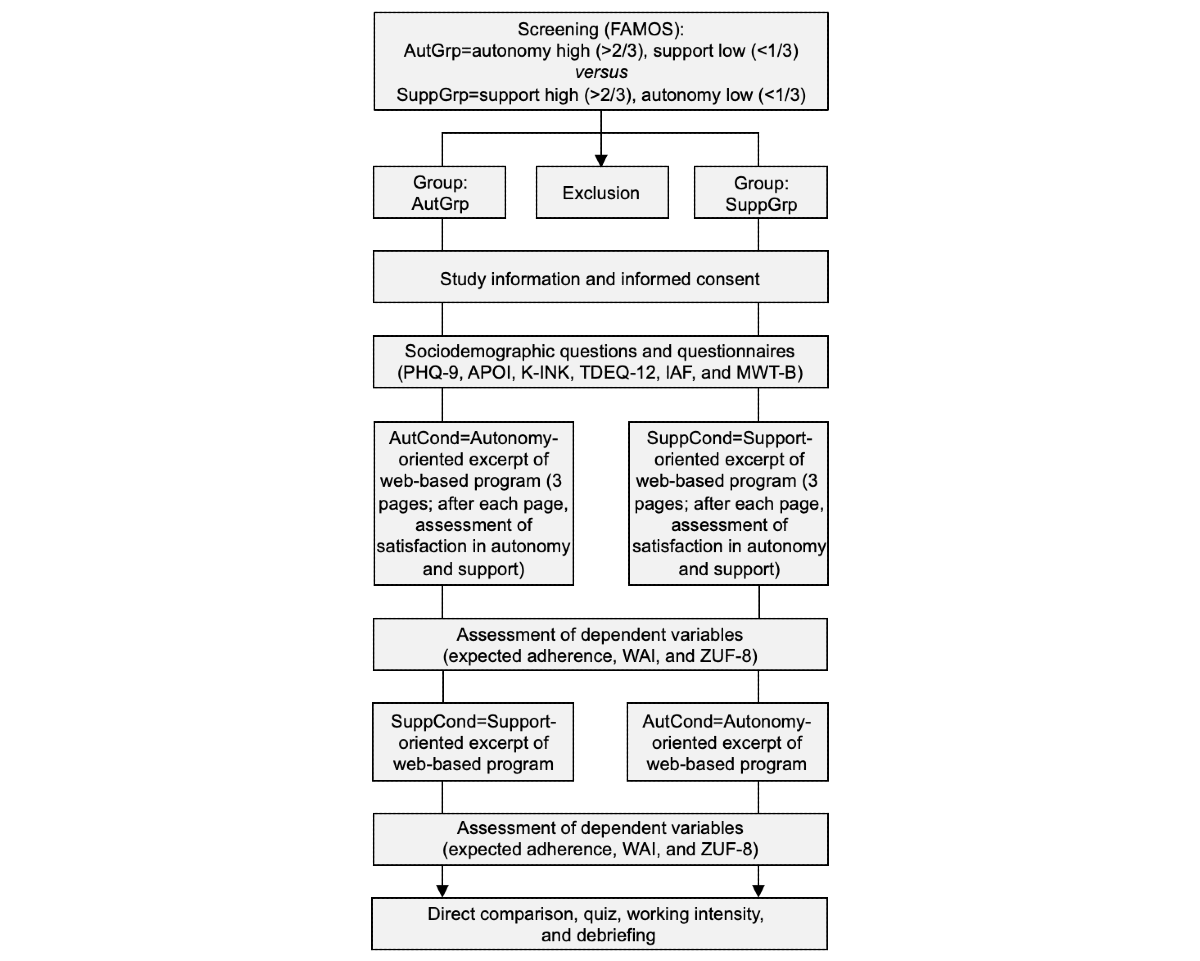
Flowchart of the assessment procedure. APOI: Attitudes Toward Psychological Online Interventions; AutCond: autonomy condition; AutGrp: autonomy group; FAMOS: Fragebogen zur Analyse Motivationaler Schemata (Inventory of Approach and Avoidance Motivation); IAF: Index of Autonomous Functioning; K-INK: Kurzversion des Inkonkruenzfragebogen (Incongruence Questionnaire, short version); MWT-B: Multiple-Choice Vocabulary Intelligence Test; PHQ-9: Patient Health Questionnaire-9, depression module; SuppCond: support condition; SuppGrp: support group; TDEQ-12: Theoretical Depressive Experiences Questionnaire, short version; WAI: Working Alliance Inventory; ZUF-8: Zufriedenheitsfragebogen (Client Satisfaction Questionnaire).

The study was performed at the University Medical Center Hamburg-Eppendorf (Germany), and the assessment was conducted on the web using the survey software Unipark (Questback). Refer to Figure 1 for a detailed procedure of the assessment.

On the first page of the excerpt of the self-help intervention, prospective participants received basic information about the study (participants were informed that they would see different versions of excerpts of an internet-based intervention that they would have to evaluate; no information on the motive adaptation was provided before the study participation) and were instructed that they would be briefly screened to determine whether they were suitable to participate in the study. In addition, to prevent fake participation by computer programs, a simple arithmetic problem had to be solved to get to the second page. Prospective participants were then screened using the Questionnaire for the Analysis of Motivation Schemas (Fragebogen zur Analyse Motivationaler Schemata [FAMOS]) [43] to form the 2 groups (high values for autonomy and low values for support vs high values for support and low values for autonomy). Candidates who did not achieve such suitable scores (refer to the *Study Design* section) on this questionnaire were excluded from further participation. The study participation then lasted approximately 1 hour, and all study participants were rewarded with a €10 (US $10.30) Amazon voucher for their participation (given only to those who passed the screening and completed the entire survey). At the end of the study, participants were informed in detail about the background and the course of the study by means of a printable information page. Subsequently, an electronic declaration of informed consent for study participation and data collection was requested.

This was followed by the administering of the questionnaires (refer to the *Measures* section) as well as the processing of both versions of the 3-page excerpts of the self-help program (refer to the *Excerpts of the Self-help Internet-Based Intervention for Depression* section) and their evaluation. After each page, the participants were asked for their motivational incongruence, mood, and arousal as well as 2 questions on how they would rate the quality and comprehensibility of the page. The order of the excerpt versions was randomized (AutCond-SuppCond or SuppCond-AutCond). After the evaluation of each excerpt, the dependent variables (expected adherence, working alliance, and treatment satisfaction) were assessed. At the end of the survey, 4 knowledge questions about the content of the self-help excerpts were asked, and the participants received a short debriefing on the rationale of the study. Subsequently, participants could enter their email address for receiving the voucher. As the email address was processed and stored independently of all other information, the study data were completely anonymous at all times. Accordingly, participants were informed that they could not subsequently request the deletion of their data. Participants were notified that they were not participating in an intervention study.

### Sample Size

The power analysis for calculating the sample size for an ANOVA was conducted using G*Power (Heinrich Heine University Düsseldorf) [44], and it revealed a sample size of n=54 to detect a medium effect of Cohen *f*=0.25, with Cronbach α=.05 and a power of 0.95.

### Recruitment

The sample was recruited through different student, psychology, and study announcement internet forums, and Facebook groups. The recruitment took place from May 16, 2019, to October 19, 2019.

### Eligibility Criteria

Those interested could participate if German was their native language, and they were aged ≥18 years. A current self-reported mental illness (eg, depression) was a criterion for exclusion. Filters were used to automatically prevent unsuitable prospective participants from participating (eg, age <18 years or self-reported mental illness).

### Ethics Approval

The local psychological ethics committee of the Center for Psychosocial Medicine of the University Medical Center Hamburg-Eppendorf approved the project (LPEK-0033; April 28, 2019). All participants provided informed consent on the web before participating in the study. The study was conducted in accordance with the Declaration of Helsinki, and the authors assert that all procedures contributing to this work comply with the ethics standards of relevant national and institutional guidelines.

### Excerpts of the Self-help Internet-Based Intervention for Depression

The excerpts that have been adapted to address the 2 motives (autonomy and support) were derived from an existing self-guided internet-based intervention with guidance in the form of text messages for depression called *MOOD* [20]. For both motives, 3 pages of the intervention were adapted to satisfy the need for autonomy (AutCond) and the need for support (SuppCond). The pages included an introduction to the program, psychoeducation on cognitive strategies and automatic thoughts, as well as information on ABC schemas. In both conditions, therapeutic support was provided in the form of text messages: the nature of the support differed in that in the SuppCond, the guide was proactive and made contact with the user, whereas in the AutGrp, the guide was available upon request, that is, the initiative came from the users. Examples of how the contents were adapted are presented in Table 1.

**Table 1:** Examples of motive-oriented adaptations.

	Autonomy condition	Support condition
Interventions as means for motive satisfaction	“The ability to become aware of automatic thoughts and thus to create more freedom of thought can be acquired in this unit.” (Module: ABC schema)	“In this way, automatic thoughts can separate you from the feeling of being connected to others, for example. The ability to become aware of automatic thoughts such as ‘He doesn’t like me anymore’ and to question them specifically is taught in this unit.”
Motive-oriented formulations	“Of course you are not told what to think. On the contrary, you remain responsible for questioning and developing your own thinking.”	“Regardless of whether it is easy for you to learn and practice the techniques in this unit or not, your personal guide will contact you regularly and ask how you are doing.”
Motive-oriented functions	Privacy settings (eg, whether guide has access to worksheets)	Accessibility guide (eg, text fields for direct questions to the guide)
Motive-oriented guidance	“If I do not receive a message from you, I assume that you will be able to manage processing of the contents of this self-help program independently.”	“Even if you do not contact me, I will check at least once a week how far you have progressed in the program and what you have entered in the worksheets to give you personal feedback with suggestions.”

### Measures

#### Overview

Data were collected on the web through the survey software Unipark. The following data were collected: motives (basis for screening and group formation; German version of the Inventory of Approach and Avoidance Motivation: FAMOS), motivational incongruence (Incongruence Questionnaire, short version [Kurzversion des Inkonkruenzfragebogen (K-INK)]), attitudes regarding web-based interventions (Attitudes Toward Psychological Online Interventions [APOI]), dependency (Theoretical Depressive Experiences Questionnaire, short version [TDEQ-12]), autonomy (Index of Autonomous Functioning [IAF]), depressiveness (Patient Health Questionnaire-9, depression module [PHQ-9]), the level of intelligence (Multiple-Choice Vocabulary Intelligence Test [MWT-B]), expected adherence, working alliance, and satisfaction with the program (Client Satisfaction Questionnaire [Zufriedenheitsfragebogen (ZUF-8)]).

#### Primary Outcome Measure

To measure the anticipated adherence, the following questions were asked about expected time investment and frequency of log-ins:

1. *How much time would you like to actively spend in the full version of the program (in hours and minutes;* optimal anticipated adherence*)?*
2. *How much time would you realistically actually spend in the full version of the program (in hours and minutes;* realistic anticipated adherence*)?*
3. *In how many of the 10 weeks would you log in to the program?*
4. *In the weeks with log-in: On average, how often would you log in to the program?*
5. *If you would still have the opportunity to log in after the 10 weeks in the program: How often would you do this in a year?*

Participants provided the answers in free-text fields, where only whole numbers could be entered.

#### Secondary Outcome Measures

##### Working Alliance Inventory

The Working Alliance Inventory (WAI) [45] is a self-rating instrument for measuring the quality of alliance based on the pantheoretical, tripartite conceptualization of the therapeutic alliance (agreement on treatment goals, agreement on the tasks of the therapy, and development of a therapeutic bond). In this study, the short version of WAI [46,47], which consists of 12 items, was used. These 12 items were adapted to correspond to an internet-based intervention. The reference was no longer to a therapist but to a guide or contact person whose availability was announced on the excerpts of the web-based program (but with whom there was no actual interaction). For each of the three subscales (goals, tasks, and bond), 4 items were rated on a 5-point Likert scale ranging from 1=*rarely* to 5=*always*. The internal consistency of the subscales is good (Cronbach α=.81-.91), and it is excellent for the total scale (Cronbach α=.90-.93 [48]).

##### Patient Satisfaction (Measured Using the ZUF-8)

The ZUF-8 [49] is the German version of the Client Satisfaction Questionnaire. This self-assessment questionnaire consists of 8 items that serve to assess patients’ treatment satisfaction (eg, psychotherapy). The 8 items can be rated on a 4-point rating scale (*excellent*, *good*, *less* *good,* and *bad*). A total score ranging from 8 to 32 can be achieved, with high scores indicating a high degree of satisfaction. The internal consistency ranges from Cronbach α=.87 to Cronbach α=.93 [50].

#### Instruments Used for the Formation and Verification of the Two Groups

##### FAMOS: Inventory of Approach and Avoidance Motivation

The self-report questionnaire FAMOS [43] captures the motives of patients undergoing psychotherapy in terms of central components of motivational schemes and was used for the formation of the 2 groups. The motives are assessed as approach motives (14 scales, eg, intimacy and attachment, status, and performance) and avoidance motives (9 scales, eg, loneliness and separation, disregard, and failure) with a total of 94 items. The items are rated on 5-point Likert scales from *not important at all* to *extremely important* or *not bad at all* to *extremely bad*. In this study, only the approach (9 items) and avoidance (10 items) scales for the 2 motives *autonomy* and *support* were assessed. The internal consistency of the individual scales varies between Cronbach α=.37 and Cronbach α=.93 [43].

##### TDEQ-12: Used to Measure Depressive Experiences

The TDEQ-12 [51], one of the short versions of the questionnaire on depressive experiences [52], is a self-reporting questionnaire that intends to distinguish between dependence and self-criticism. The questionnaire consists of 12 items that are scored on a 7-point Likert scale ranging from 1=*strongly disagree* to 7=*strongly agree*. The questionnaire has satisfactory psychometric properties. The dependency factor was made up of 5 items that had internal consistency with a Cronbach α=.77, whereas the internal consistency of the self-criticism factor consisting of 7 items had a Cronbach α=.78 [53]. In our study, only the 5 items assessing dependency were used to check whether the 2 groups differed significantly in dependency.

##### IAF: Used to Measure Trait Autonomy

The IAF [54] is a self-rating questionnaire to measure trait autonomy based on three theoretically derived subscales assessing authorship or self-congruence, interest-taking, and low susceptibility to control. The questionnaire consists of 5 items per scale that can be answered on a 5-point rating scale (1=*not at all true*, 2=*a bit true*, 3=*somewhat true*, 4=*mostly true*, and 5=*completely true*). The internal consistency of the scale is good (Cronbach α=.82 [54]). The IAF was used to check whether the 2 groups differed significantly in autonomy.

#### Instruments Used for Testing Group Comparability

##### APOI: Used to Measure Attitudes Toward Psychological Web-Based Interventions

The APOI [55] is a self-assessment questionnaire for assessing attitudes toward psychological web-based interventions covering four dimensions: (1) skepticism and risk perception, (2) trust in therapeutic efficacy, (3) perception of deficits in mechanization, and (4) perception of advantages of anonymity. It consists of 16 items that can be rated on a 5-point rating scale ranging from 1=*do not agree at all* to 5=*fully agree*. A higher total score indicates a more positive attitude toward psychological web-based interventions. All 4 dimensions are equally weighted. The APOI’s internal consistency has a Cronbach α=.77 [55].

##### K-INK: Used to Measure Incongruence

Incongruence (unsatisfactory fit of motivational goal and actual experience) was assessed with the short version of the INK [56], a self-rating questionnaire with originally 94 items on 14 approach scales and 9 avoidance scales. The INK has satisfactory reliability (Cronbach α=.72-.87) and validity. Item contents and scale structure are derived from the FAMOS. Although the FAMOS measures the intensity of motives (importance or being bad), the INK measures the degree of insufficient implementation of these motives (satisfaction with the implementation of approach motives or the occurrence of avoidance motives). The short version (K-INK) contains only the 23 items with the highest item total correlations. Answers can be given on a 5-point Likert scale ranging from 1=*much too little* to 5=*completely sufficient*.

##### PHQ-9: Used to Measure Depressive Symptoms

Depressive symptoms were assessed with the PHQ-9 [57], which is a self-rating questionnaire that consists of 9 items on depressive symptoms over the past 2 weeks. The items can be answered on a 4-point Likert scale ranging from 0=*not at all* to 3=*nearly every day*. The questionnaire can assist the diagnosis of major depression according to Diagnostic and Statistical Manual of Mental Disorders, Fourth Edition, criteria. Its internal consistency ranges from Cronbach α=.86 to Cronbach α=.89 [58].

##### MWT-B: Used to Measure General Intelligence Levels

The MWT [59] is a performance test to measure general intelligence levels, specifically crystallized intelligence. The test includes 2 versions—A and B. Both versions consist of a total of 37 lines with 5 terms each. In each line only 1 of these 5 terms is a real word; this is the one to be found out and marked by the participant. The other terms are fictitious constructions. Test results correlate quite well with the global IQ of healthy adults (median of *r*=0.72 [59]). In our study, we randomly presented each participant with only 1 item (a line of 5 terms) to exclude participants whose intelligence level was too low per the exclusion criteria.

### Statistical Analyses

Group differences in sociodemographic characteristics and assessed questionnaires were determined using Welch 2-tailed *t* tests and chi-square tests. The Shapiro-Wilk test was used to test for normality of data. Logarithmic (log) transformation was applied for skewed data. The main hypothesis was tested using an ANOVA with repeated measurements (within-subject: motive orientation and between-subject: motive). Multiple comparisons were adjusted with Bonferroni correction. Within-group differences were analyzed with paired sample *t* tests and between-group differences with independent sample *t* tests. Correlative relationships were analyzed using Pearson correlation coefficients.

## Results

### Sample Characteristics

In total, 55 participants were included in the analyses. No participant was excluded from the study on account of intelligence levels (based on the MWT-B). Sample characteristics are depicted in Table 2. The participants’ mean age was 27.27 (8.18) years. The majority of the participants were women (47/55, 85%), and almost half were single (27/55, 49%). The 2 groups (AutGrp: 27/55, 49%, and SuppGrp: 28/55, 51%) did not differ in sociodemographic characteristics, depressive episodes in the past (lifetime), current depressive symptoms (PHQ-9), attitudes toward web-based interventions (APOI), or motivational incongruence (K-INK).

The formation of 2 groups based on the screening with the FAMOS questionnaire worked very well. As intended, the 2 groups differed significantly in their responses to independent questionnaires of autonomy (IAF; *P*=.01; Cohen *d*=–0.786, 95% CI –1.335 to –0.237) and dependence (TDEQ-12; *P<*.001; Cohen *d*=1.321, 95% CI 0.738-1.905) with large effect sizes. Thus, the AutGrp had high levels of autonomy and low levels of dependence compared with the SuppGrp, and the check of the group formation can be deemed successful.

**Table 2:** Sociodemographic characteristics of the sample (N=55).

	Autonomy group (n=27)	Support group (n=28)	*t* test (*df*)	Chi-square (*df*)	*P* value
Age (years), mean (SD)	28.48 (9.44)	26.11 (6.71)	1.07 (53)	N/A^a^	.29
Sex (female), n (%)	22 (81)	25 (89)	N/A	0.67 (2)	.41
Marital status (single), n (%)	15 (56)	12 (43)	N/A	0.89 (3)	.35
School education (years), mean (SD)	12.11 (0.80)	12.29 (1.08)	–0.68 (53)	N/A	.50
Past depression, n (%)	4 (15)	6 (21)	N/A	0.40 (1)	.53
Autonomy (IAF^b^), mean (SD)	4.13 (0.59)	3.65 (0.63)	2.94 (53)	N/A	.01
Dependence (TDEQ-12^c^), mean (SD)	2.67 (1.23)	4.59 (1.64)	–4.92 (53)	N/A	*<*.001
Depressive syndrome (PHQ-9^d^), mean (SD)	13.54 (3.87)	15.03 (4.41)	–1.33 (53)	N/A	.19
Attitudes toward web-based interventions (APOI^e^), mean (SD)	52.26 (8.14)	49.29 (7.67)	1.39 (53)	N/A	.17
Motivational incongruence (K-INK^f^), mean (SD)	2.01 (0.46)	2.27 (0.66)	–1.72 (53)	N/A	.09

^a^N/A: not applicable.

^b^IAF: Index of Autonomous Functioning.

^c^TDEQ-12: Theoretical Depressive Experiences Questionnaire, short version.

^d^PHQ-9: Patient Health Questionnaire-9, depression module.

^e^APOI: Attitudes Toward Psychological Online Interventions.

^f^K-INK: Kurzversion des Inkonkruenzfragebogen (Incongruence Questionnaire, short version).

### Manipulation Checks

**Figure 2:**
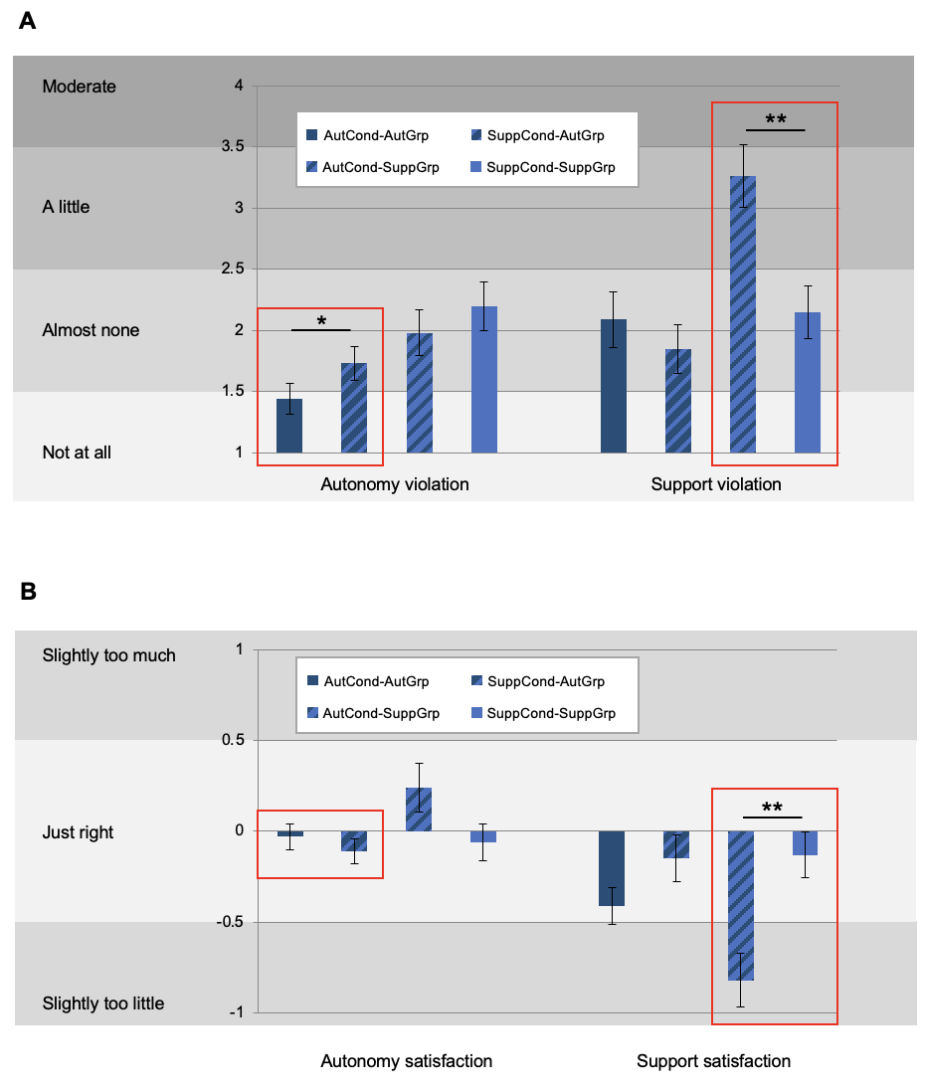
Manipulation checks. (A) Autonomy and support violation per group and condition. (B) Autonomy and support satisfaction per group and condition. AutCond: autonomy condition; AutGrp: autonomy group; SuppCond: support condition; SuppGrp: support group. *P<.05, **P<.001.

The violation and satisfaction of the 2 motives *being autonomous* and *being supported* for the different fits of group and condition are presented in Figure 2. Focusing on the incongruent and congruent fits (ie, mismatch and match) for the AutGrp in autonomy violation, the incongruent fit SuppCond-AutGrp reported a higher autonomy violation (mean 1.73, SD 0.72) than the congruent fit AutCond-AutGrp (mean 1.44, SD 0.65; t_27_=2.36; *P*=.03), which is in line with our expectations. The results of the interaction effect of the repeated measures ANOVA showed that all 4 combinations did not differ significantly in autonomy violation (*F*_1,53_=0.06; *P*=.80). However, the main effects for condition (*F*_1,53_=4.95; *P*=.03) and group (*F*_1,53_=5.90; *P*=.02) were significant. Overall, the SuppCond (mean 1.97, SE 0.12) and the SuppGrp (mean 2.09, SE 0.15) experienced higher levels of autonomy violation than the AutCond (mean 1.71, SE 0.12) and the AutGrp (mean 1.57, SE 0.15).

For support violation, AutCond-SuppGrp reported a higher support violation (mean 3.26, SD 1.36) than the congruent fit SuppCond-SuppGrp (mean 2.15, SD 1.14; t_28_=4.50; *P*<.001), which is also consistent with our expectations. For support violation, the interaction effect was significant (*F*_1,53_=7.37; *P*=.009), as were both the main effects for condition (*F*_1,53_=17.44; *P*<.001) and group (*F*_1,53_=7.12; *P*=.01). For condition, support violation was higher in the AutCond (mean 2.67, SE 0.17) than in the SuppCond (mean 2.00, SE 0.15). However, for group, support violation was higher in the SuppGrp (mean 1.97, SE 0.20) than in the AutGrp (mean 2.71, SE 0.19). The interaction term stems from a significant difference among the conditions in the SuppGrp (t_27_=5.00; *P*<.001) and a nonsignificant difference among the conditions in the AutGrp (t_26_=1.01; *P*=.32).

Regarding autonomy satisfaction, as expected, autonomy in the incongruent fit SuppCond-AutGrp was *slightly too little* (mean *–*0.11, SD 0.36), and the congruent fit AutCond-AutGrp reported an autonomy satisfaction that was almost *just right* (mean *–*0.03, SD 0.37). However, this difference was not significant (t_27_=0.83; *P*=.41). The interaction effect (*F*_1,53_=2.21; *P*=.14) and the main effect for group (*F*_1,53_=1.78; *P*=.19) for autonomy satisfaction were not significant, but the main effect for condition was significant (*F*_1,53_=4.95; *P*=.03). Specifically, satisfaction regarding autonomy was closer to *just right* in the SuppCond (mean –0.09, SE 0.06) than in the AutCond (mean 0.10, SE 0.08).

Finally, for support satisfaction, the incongruent fit AutCond-SuppGrp (mean *–*0.82, SD 0.79) had the lowest ratings (*slightly too little* support). In the congruent fit SuppCond-SuppGrp, the rating was the closest to *just right* (mean *–*0.06, SD 0.54). This difference was significant (t_28_=4.34; *P*<.001). The interaction effect here was significant (*F*_1,53_=4.88; *P*=.03), as was the main effect for condition (*F*_1,53_=3.74; *P*<.001); however, the effect for group was not significant (*F*_1,53_=1.97; *P*=.17). Overall, the satisfaction regarding support was closer to *just right* in the SuppCond (mean *–*0.14, SE 0.08) than in the AutCond (mean *–*0.61, SE 0.09). The significant interaction term stems from a significant difference between the groups in the AutCond (t_53_=2.27; *P*=.03) and a nonsignificant difference between the groups in the SuppCond (t_53_=0.10; *P*=.92).

### Anticipated Adherence

**Figure 3:**
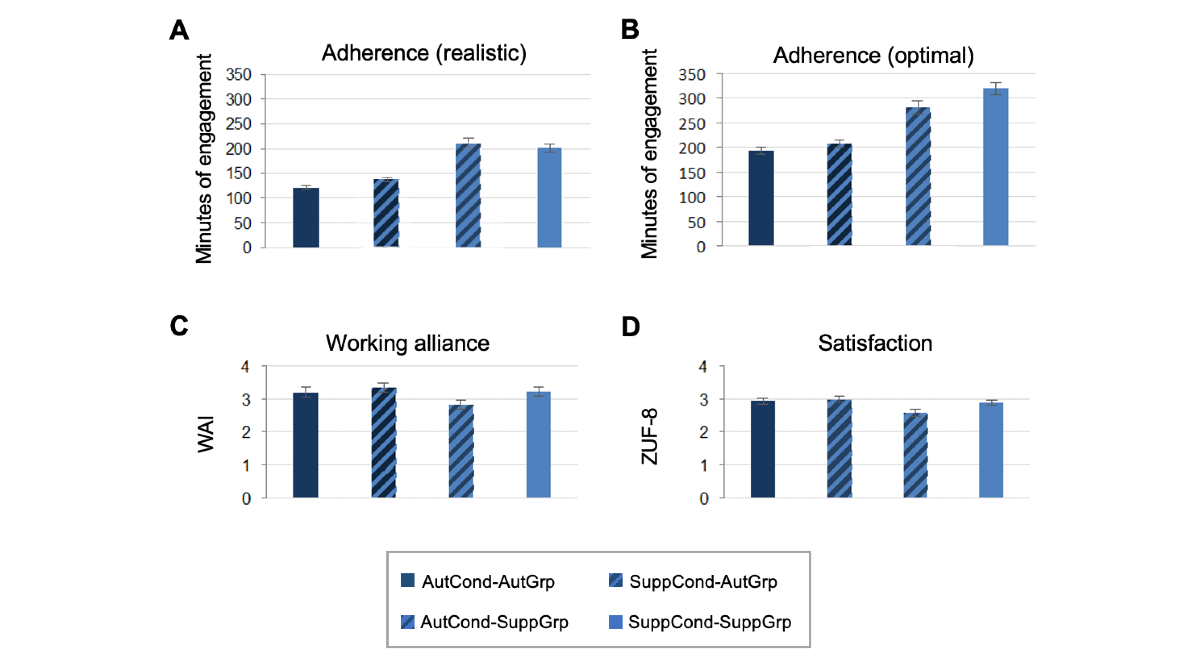
(A) Anticipated realistic adherence per group and condition. (B) Anticipated optimal adherence per group and condition. (C) Working alliance per group and condition. (D) Satisfaction per group and condition. AutCond: autonomy condition; AutGrp: autonomy group; SuppCond: support condition; SuppGrp: support group; WAI: Working Alliance Inventory; ZUF-8: Zufriedenheitsfragebogen (Client Satisfaction Questionnaire).

The results of the anticipated adherence (in anticipated minutes spent with the program; optimal and realistic), working alliance, and satisfaction are shown in Table 3 and Figure 3. The highest anticipated adherence (both optimal and realistic) was found for the combinations SuppCond-SuppGrp (optimal adherence: mean 319.39, SD 333.60, and realistic adherence: mean 201.82, SD 230.96; refer to Table 3 for all log-transformed data) and AutCond-SuppGrp (optimal adherence: mean 280.29, SD 356.16, and realistic adherence: mean 209.32, SD 305.01); the lowest anticipated adherence was found for the combination AutCond-AutGrp (optimal adherence: mean 193.59, SD 180.82; realistic adherence: mean 121.15, SD 97.93). The results of the interaction effect of the repeated measures ANOVA showed that the combinations did not significantly differ for anticipated adherence (log–optimal adherence: *F*_1,53_=0.88; *P*=.35, and log–realistic adherence: *F*_1,53_=0.59; *P*=.45). In addition, no main effects for group were found (log–optimal adherence: *F*_1,53_=0.32; *P*=.86, and log–realistic adherence: *F*_1,53_=0.001; *P*=.98). For condition, a main effect was present for optimal anticipated adherence (*F*_1,53_=6.49; *P*=.01), with higher ratings in the SuppCond (mean 5.07, SE 0.14) than in the AutCond (mean 4.89, SE 0.15), but a main effect was not present for realistic anticipated adherence (*F*_1,53_=2.04; *P*=.16).

The results of the paired sample *t* tests indicated significant within-group differences for optimal anticipated adherence in the SuppGrp (t_27_=3.00; *P*=.006). No significant within-group differences could be found for realistic anticipated adherence in the AutGrp.

Several correlative relationships between motive satisfaction or dissatisfaction and anticipated (optimal and realistic) adherence could be found. For the congruent person-program fit AutCond-AutGrp, support satisfaction negatively correlated with optimal anticipated adherence (*r*=–0.40; *P*=.04) and, in a matching result, support dissatisfaction positively correlated with optimal anticipated adherence (*r*=0.43; *P*=.02). In addition, for the incongruent fit SuppCond-AutGrp, support satisfaction negatively correlated with optimal anticipated adherence (*r*=–0.59; *P*=.001), and in another matching result, support dissatisfaction negatively correlated with optimal anticipated adherence (*r*=0.58; *P*=.002). For realistic adherence, in the congruent person-program fit AutCond-AutGrp, autonomy satisfaction correlated with realistic anticipated adherence (*r*=0.46; *P*=.02).

**Table 3:** Anticipated adherence (optimal and realistic) shown as expected time (in minutes) spent with the program; working alliance was measured with the Working Alliance Inventory (WAI), and satisfaction with the program was measured with the Client Satisfaction Questionnaire (Zufriedenheitsfragebogen [ZUF-8]; N=55).

Condition and variables		Autonomy group (n=27)		Support group (n=28)	
		Values, mean (SD)	Log-transformed mean (SD)	Values, mean (SD)	Log-transformed mean (SD)
**Autonomy condition**					
	Adherence (optimal)	*193.59 (180.82)* ^a^	*4.89 (0.90)*	280.29 (356.16)	4.88 (1.27)
	Adherence (realistic)	*121.15 (97.93)*	*4.52 (0.84)*	209.32 (305.01)	4.43 (1.35)
	WAI	*3.19 (0.80)*	*N/A* ^b^	2.82 (0.73)	N/A
	ZUF-8	*2.93 (0.53)*	*N/A*	2.56 (0.59)	N/A
**Support condition**					
	Adherence (optimal)	207.85 (182.45)	5.01 (0.81)	*319.39 (333.60)*	*5.13 (1.25)*
	Adherence (realistic)	138.00 (128.95)	4.59 (0.83)	*201.82 (230.96)*	*4.67 (1.20)*
	WAI	3.34 (0.79)	N/A	*3.21 (0.66)*	*N/A*
	ZUF-8	2.97 (0.49)	N/A	*2.88 (0.45)*	*N/A*

^a^Italics indicate congruence (normal font indicates incongruence).

^b^N/A: not applicable.

### Working Alliance and Satisfaction

For working alliance (with the program and a guide whose availability was announced), neither a significant interaction effect (*F*_1,53_=2.12; *P*=.15) nor a significant main effect for group (*F*_1,53_=1.84; *P*=.18) was shown, but a significant main effect for condition was present (*F*_1,53_=11.23; *P*=.001). Working alliance was descriptively highest for the combination SuppCond-AutGrp (mean 3.34, SD 0.79) and lowest for the combination AutCond-SuppGrp (mean 2.82, SD 0.73). Likewise, no significant interaction effect was found for satisfaction (*F*_1,53_=3.48; *P*=.07). Here, the main effect for group bordered on significance (*F*_1,53_=3.77; *P*=.06), whereas the main effect for condition was significant (*F*_1,53_=5.56; *P*=.02). Satisfaction was significantly higher in the SuppCond (mean 2.92, SE 0.06) than in the AutCond (mean 2.75, SE 0.08). Satisfaction was numerically highest for SuppCond-AutGrp (mean 2.97, SD 0.49) and lowest for AutCond-SuppGrp (mean 2.56, SD 0.59).

The paired sample *t* tests showed significant within-group differences for working alliance (t_27_=3.20; *P*=.003) and satisfaction (t_27_=2.86; *P*=.008) for the SuppGrp, with higher scores in the matching case than in the mismatch case, but not for the AutGrp. Independent sample *t* tests revealed significant differences for satisfaction in the AutCond between both groups (t_53_=2.44; *P*=.02), with the AutGrp being more satisfied than the SuppGrp, but no significant differences were revealed for any other parameter.

Support satisfaction correlated with treatment satisfaction measured with the ZUF-8 for the congruent fit AutCond-AutGrp (*r*=0.60; *P*=.001) as well as for the incongruent fit AutCond-SuppGrp (*r*=0.69; *P*<.001) and, in a matching result, support dissatisfaction negatively correlated with treatment satisfaction for the same fits (AutCond-AutGrp: *r*=–0.59; *P*=.001; AutCond-SuppGrp: *r*=–0.80; *P*<.001). Support satisfaction correlated with working alliance for the congruent fit AutCond-AutGrp (*r*=0.53; *P*=.004) and the incongruent fit AutCond-SuppGrp (*r*=0.58; *P*=.001), whereas support dissatisfaction negatively correlated with working alliance for AutCond-AutGrp (*r*=–0.50; *P*=.008) and AutCond-SuppGrp (*r*=–0.72; *P*<.001).

## Discussion

### Overview

Low adherence in self-guided internet-based interventions for depressive and other mental disorders is a significant issue and might be optimized by adapting the content and the context of the interventions to the personal needs and motives of the user, resulting in more engaging and less aversive experiences. In this nonclinical experimental study, the 2 motives *being autonomous* and *being supported* were used to test the hypothesis that a better motivational person-program fit is associated with less motivational incongruence and higher anticipated adherence. The formation of 2 groups was successful; the manipulation check suggested that a congruent person-program fit is associated with lower subjective motive violation and partly higher motive satisfaction (not for autonomy satisfaction) compared with a noncongruent fit, but the main hypotheses were only partially supported. This study represents a first attempt to experientially examine the adaptation of internet-based interventions to individuals’ motives as a tool to increase adherence, working alliance, and satisfaction with the interventions. This is a novel way to personalize internet-based interventions, inspired by theories and findings from motive-oriented therapeutic relationship building in face-to-face therapies [36,37,41,42].

### Manipulation

The formation of 2 groups based on the screening with the FAMOS was successful and revealed significant differences between the 2 groups in autonomy and dependence, with large effect sizes in independent measures. The manipulation (adapting the excerpts of the intervention and the form of the intervention to the 2 opposed motives) can be regarded as almost successful. The incongruent fits reported a significantly higher violation of the 2 motives than the congruent fits. Regarding the motive satisfaction, the congruent fit also resulted in a significantly higher support satisfaction (closest to *just right*) than the incongruent fit. However, this was not the case for autonomy satisfaction (*P*=.41). The requirements for the experimental setup can therefore be considered as fulfilled for autonomy and support violation and support satisfaction but not for autonomy satisfaction. It may have been a decisive factor that the motives *being autonomous* and *being supported* were only explicitly captured by a questionnaire. It is conceivable that these constructs can be better assessed using implicit measures [60,61]. In addition, the autonomy-granting condition might have had a less strong effect than the support-granting condition, which is another explanation for the lack of differences in autonomy satisfaction.

### Main Results

Contrary to our expectations, there was no significant interaction between group and condition regarding the self-reported hypothetical expectation of the use of the *full version* of the program. Within-group differences for optimal anticipated adherence were significant in the SuppGrp. Thus, participants who described themselves as needing support reported that they would be more adherent to a program that matches their needs, corroborating the main hypotheses of this paper. However, surprisingly, this was not the case for the AutGrp. It therefore seems that the SuppGrp was more satisfied with the program overall (in contrast to the AutGrp) and that a congruent motive orientation for this group (SuppGrp) resulted in a higher anticipated adherence than an incongruent motive orientation. An explanation for these results could be that in the SuppCond, the texts directly referred to guidance in the form of personal therapeutic support (eg, “Personal guide will contact you regularly and ask how you are doing” and “Even if you do not contact me, I will check at least once a week how far you have progressed in the program and what you have entered in the worksheets to give you personal feedback with suggestions”). From a large base of studies, we know that guidance has a beneficial effect on adherence and effectiveness [2,8,15,62]. It is conceivable that the expectation of therapeutic guidance alone has a greater effect on anticipated adherence than more supportive language or wording alone.

Although for working alliance (measured using the WAI) neither a significant interaction effect nor a significant main effect for group was shown, there was a significant main effect for condition: the SuppCond was accompanied by higher working alliance. Similarly, no significant interaction and group effects were found for satisfaction with the program (measured using the ZUF-8), whereas the main effect for condition was significant: the supportive program was expected to be more satisfying. As for anticipated adherence, within-group differences (match vs mismatch) for working alliance and satisfaction were significant for the SuppGrp, again corroborating one of our main hypotheses, but not for the AutGrp.

An explanation for the nonsignificant within-group results in the AutGrp might be that the manipulation for autonomy satisfaction did not completely work out. Therefore, the results must be considered with caution.

### Limitations

This study includes several limitations. An important limitation is that adherence to the internet-based intervention was not measured as true adherence on a behavioral level but as time in anticipated minutes working with the program. Thus, both the validity of the measures and the generalizability of the associated findings can be partially questioned. However, treatment expectations are known to have a major impact because they are among the most important predictors of outcomes [63,64]. Furthermore, the sample was not a clinical sample and was recruited mainly through student forums or psychology groups, again diminishing the generalizability of the findings. Particularly, the lack of distress and treatment motivation in this nonclinical sample is likely to have led to smaller effects (ie, because participants did not experience distress, they may not have felt it necessary to spend much time with the web-based program), which the study was not powered to detect. Taken together, the successful formation of groups apparently with matching and mismatching program versions was strong enough to induce motive satisfaction and violation, but the impact on more distal measures such as anticipated adherence might have been underestimated in this nonclinical sample. An additional shortcoming is that no control condition (evaluating a program with no motive adaptation) was included in the study, resulting in a limited validity of the results, and the motives were assessed only explicitly with the FAMOS and not implicitly.

### Conclusions

Our study should be seen as a first proof of concept from which programs could now be adapted and evaluated for other motives. Despite the inconclusive results regarding autonomy, the study gives reason to assume that motive orientation could have a positive impact on adherence, working alliance, and satisfaction in internet-based interventions for depression and other mental disorders. This should be investigated in future randomized controlled trials with clinical samples that allow assessment of the actual (and not only the anticipated) adherence, working alliance, and treatment satisfaction.

## Abbreviations

APOI: Attitudes Toward Psychological Online Interventions
AutCond: autonomy condition
AutGrp: autonomy group
FAMOS: Fragebogen zur Analyse Motivationaler Schemata
IAF: Index of Autonomous Functioning
K-INK: Kurzversion des Inkonkruenzfragebogen
MWT-B: Multiple-Choice Vocabulary Intelligence Test
PHQ-9: Patient Health Questionnaire-9, depression module
SuppCond: support condition
SuppGrp: support group
TDEQ-12: Theoretical Depressive Experiences Questionnaire, short version
WAI: Working Alliance Inventory
ZUF-8: Zufriedenheitsfragebogen
